# Cribriform Patterned Lesions in the Prostate Gland with Emphasis on Differential Diagnosis and Clinical Significance

**DOI:** 10.3390/cancers14133041

**Published:** 2022-06-21

**Authors:** Maria Destouni, Andreas C. Lazaris, Vasiliki Tzelepi

**Affiliations:** 1 Department of Cytopathology, Hippokrateion General Hospital of Athens, 11527 Athens, Greece; med5673@upnet.gr; 2 First Department of Pathology, School of Medicine, The National and Kapodistrian University of Athens, 11527 Athens, Greece; alazaris@med.uoa.gr; 3 Department of Pathology, School of Medicine, University of Patras, 26504 Patras, Greece

**Keywords:** prostate cancer, intraductal carcinoma, cribriform carcinoma, Gleason Score, prognostic grade group, prognosis

## Abstract

**Simple Summary:**

A cribriform structure is defined as a continuous proliferation of cells with intermingled lumina. Various entities may have a cribriform morphology within the prostate gland, ranging from normal, to benign, to borderline and even to malignant lesions. This review summarizes the morphologic features of entities that have a cribriform morphology within the prostate gland, with an emphasis on their differential diagnosis, molecular profile and clinical significance. The basic aim is to assist the pathologist with challenging and controversial cases and inform the clinician on the clinical implications of cribriform morphology.

**Abstract:**

Cribriform glandular formations are characterized by a continuous proliferation of cells with intermingled lumina and can constitute a major or minor part of physiologic (normal central zone glands), benign (clear cell cribriform hyperplasia and basal cell hyperplasia), premalignant (high-grade prostatic intraepithelial neoplasia), borderline (atypical intraductal cribriform proliferation) or clearly malignant (intraductal, acinar, ductal and basal cell carcinoma) lesions. Each displays a different clinical course and variability in clinical management and prognosis. The aim of this review is to summarize the current knowledge regarding the morphological features, differential diagnosis, molecular profile and clinical significance of the cribriform-patterned entities of the prostate gland. Areas of controversy regarding their management, i.e., the grading of Intaductal Carcinoma, will also be discussed. Understanding the distinct nature of each cribriform lesion leads to the correct diagnosis and ensures accuracy in clinical decision-making, prognosis prediction and personalized risk stratification of patients.

## 1. Introduction

Glandular structures within the prostate may assume a cribrifrom morphology as a major or minor part of physiological, non-neoplastic and neoplastic processes. Cribriform is derived from the Latin word *cribrum*, which means sieve and is used to describe something that is pierced with small holes. In histology, cribriform is defined as a continuous proliferation of cells with intermingled lumina. That means that there are cells lying one next to another with round or elongated holes interspaced among them at various intervals. Normal prostate glands and benign (clear cell cribriform hyperplasia and basal cell hyperplasia), premalignant (high-grade prostatic intraepithelial neoplasia), borderline (atypical intraductal cribriform proliferation) or clearly malignant (intraductal, acinar, ductal and basal cell carcinoma) lesions can present with a cribriform morphology. These entities display a different clinical course and variability in clinical management and prognosis. Thus, cribriform morphology can cause a diagnostic challenge for the pathologist. In addition, even though cribriform morphology in benign entities is probably the result of cells with glandular differentiation pilling up within a pre-existing space (duct), in malignant entities, it may be a purposeful architectural pattern that offers a survival advantage to cancer cells, as it has been associated with adverse prognosis and therapy resistance.

Overall, the aim of this review is to summarize the current knowledge regarding the morphological features of cribriform formations within the prostate gland, their differential diagnosis and the utility of immunohistochemical markers (i.e., PTEN loss and ERG expression) in establishing an accurate diagnosis. Areas of controversy regarding the clinical significance and/or management of cribriform entities will also be discussed. In Table 1, the most important morphologic and molecular features of these entities are summarized.

## 2. Benign Cribriform Formations

The cribriform pattern is commonly observed in normal tissue and benign processes of the prostate gland. Although these entities can become potential mimickers of atypical and even malignant lesions, under no circumstances must they be confused with cancer, as they have no significant clinical implications. Their features are discussed below.

### 2.1. Normal Cribriform Glands

**Figure 1:**
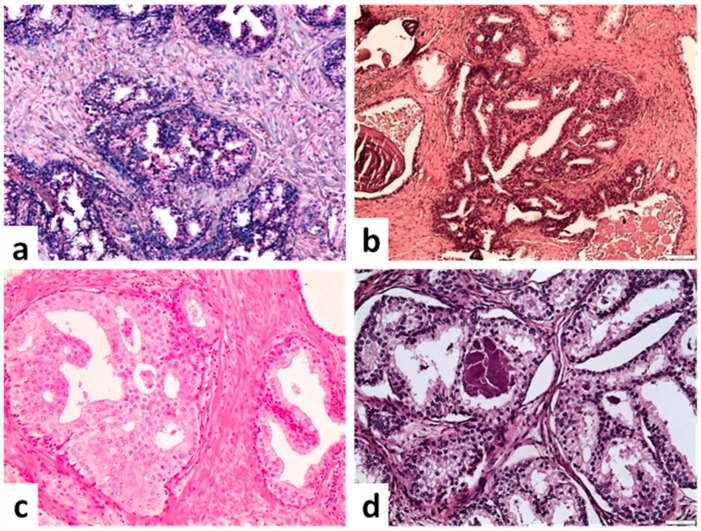
Benign cribriform formations. (**a**) Glands in the base of the prostate with luminal tufting (upper part) and nuclear pseudostratification. A cribriform architectural pattern is observed, but lack of cytologic atypia and presence of basal cell layer excludes malignancy (×100). (**b**) Basal cell hyperplasia with a cribriform pattern (×100). (**c**) Hyperplastic cribriform gland lacking cytologic atypia and displaying abundant pale to clear cytoplasm similar to acinar cells of adjacent prostatic gland (right side) (×200). (**d**) Clear cell cribriform hyperplasia with bland monotonous nuclei (×200) (scale bar is 50 μm).

The epithelial tissue of the central zone of the prostate gland near its base can form cribriform glandular structures [1]. These structures are composed of complex papillary epithelium with cribriform morphology, Roman Arches and epithelial bridges [1,2] lined by columnar, pseudostratified epithelial cells with granular eosinophilic cytoplasm and a small round nucleus. Of note, the cytologic atypia or prominent nucleoli are consistently absent. In addition, the presence of an intact and frequently prominent basal cell layer is characteristic and can help to identify the benign nature of the lesion (Figure 1a). This totally normal cribriform morphology has been well understood and is easily distinguishable from other cribriform lesions of the prostate gland—albeit, its differential diagnosis may be more challenging in biopsies.

### 2.2. Basal Cell Hyperplasia

Basal cell hyperplasia constitutes a focal, nodular process composed of an expansion of basal cells within the acini of prostate gland [3]. Small clusters of proliferating round basal cells that are two or more layers in thickness are formed within a compressed stroma. These cell clusters may grow in a solid, cystically dilated or cribriform pattern [3,4]. The cribriform architectural pattern is composed of irregular round luminal spaces. Cytologically, basal cells have scant cytoplasm with hyperchromatic nuclei and lack cytologic atypia (Figure 1b). Immunohistochemically, the cells in basal cell hyperplasia are positive for basal cell markers (p63, HMWCK) and negative for AMACR [3]. Basal cell hyperplasia is not associated with an adverse prognosis or a higher risk of prostate cancer.

### 2.3. Clear Cell Cribriform Hyperplasia

Clear cell cribriform hyperplasia represents a rare variant in the histologic spectrum of benign nodular prostatic hyperplasia (BPH), is typically located in the transitional zone and is composed of a nodular cluster of medium- and large-sized acini with cribriform morphology. The hyperplastic epithelial cells have a pale-to-clear granular cytoplasm, and their nuclei lack cytologic atypia or prominent nucleoli. A clearly visible basal cell layer is always present (Figure 1c,d). Cribriform hyperplasia does not constitute a risk factor for prostate cancer and the treatment is the same as in BPH.

As far as a differential diagnosis is concerned, the normal and benign cribriform formations described above may be confused with cribriform adenocarcinoma, intraductal carcinoma (IDC) and high-grade prostatic intraepithelial neoplasia (HGPIN), especially when a prostate needle biopsy specimen is evaluated. In this case, the benign cytologic features (absence of prominent nucleoli and lack of cytologic atypia) and the presence of a prominent and continuous basal cell layer support the diagnosis of a benign entity. In addition, the similarity in cellular morphology between the cribriform structure in question and adjacent normal acini is helpful to confirm the non-malignant nature of these cribriform glands.

## 3. Premalignant Cribriform Lesions

### High-Grade Prostatic Intraepithelial Neoplasia (HGPIN)

**Figure 2:**
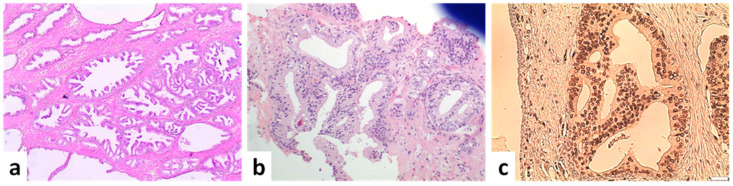
Preneoplastic and borderline neoplastic cribriform lesions. (**a**) Multiple foci of high-grade PIN (×40). (**b**) Atypical intraductal cribriform proliferation in a biopsy. Proliferation is present in <50% of the gland, located at the edge of the core and not accompanied by atypia or necrosis (×100). (**c**) Atypical intraductal cribriform proliferation. An expanded gland with a loose cribriform architecture is observed. Immunohistochemical expression of the marker ERG is suggestive of the malignant nature of neoplastic cells but is not enough to change the diagnosis to IDC (×100) (scale bar is 50 μm).

HGPIN is regarded as a precursor lesion to invasive adenocarcinoma. This lesion shares phenotypic and genetic alterations with invasive carcinoma but lacks invasion into the fibromuscular stroma [5]. In HGPIN, luminal epithelial growth takes place within ducts/glands of normal size without expansion of their lumina. This lack of expansion of the involved glandular spaces is contrasted to intraductal carcinoma which typically involves expanded acini and ducts. Cytologic atypia is present and includes nuclear enlargement, hyperchromatic nuclei, prominent nucleoli, slightly amphophilic cytoplasm, and nuclear stratification. Necrosis is absent and the basal cell layer is preserved, although it can be fragmented (Figure 2a). Four main growth patterns were initially described in HGPIN: tufting, micropapillary, cribriform, and flat [6]. However, the cribriform pattern is no longer considered acceptable in HGPIN. Lesions with a loose cribriform pattern and no necrosis or significant pleomorphism are considered atypical cribriform lesions (see below), whereas lesions with dense cribriform pattern and/or necrosis or nuclear enlargement/pleomorphism are diagnosed as IDC.

The distinction of HGPIN from IDC is particularly important, especially in biopsies, as the two entities have different clinical implications and a different subsequent approach is followed for each of them [7,8]. The distinction is usually straightforward because, as mentioned above, dense cribriform architecture, nuclear pleomorphism and necrosis are not features that are consistent with the diagnosis of HGPIN. Gland expansion, the involvement of >6 glands, presence of an irregular contour and identification of a biphasic cell population favor IDC [8,9]. In cases where it is difficult to distinguish HGPIN from IDC morphologically (i.e., in flat lesions with moderate nucleomegaly), immunohistochemistry can be of use; ERG expression and PTEN loss is observed in IDC, whereas ERG is less frequently expressed and PTEN expression is preserved in HGPIN [7,10,11,12].

## 4. Cribriform Lesions with Borderline Clinical Significance

### Atypical Intraductal Cribriform Proliferation (AIDCP)

Atypical intraductal cribriform proliferation (AIDCP) is a relatively new term that describes a cribriform lesion with a phenotype more atypical than HGPIN but without meeting all the diagnostic features of IDC. The characteristics of AIDCP on needle biopsies include (a) loose cribriform lumen-spanning architecture beyond that of HGPIN, but lacking significant nuclear pleomorphism or necrosis to meet the criteria for IDC; (b) atypical nuclei with significant pleomorphism but insufficient for a diagnosis of IDC; and/or (c) dense cribriform or solid proliferation of atypical cells partially present in large ducts on the edge of core biopsy specimens (Figure 2b,c) [10]. Basal cells are retained in AIDCP.

The differential diagnosis of AIDCP from HGPIN is usually straightforward, as cribriform architecture is no longer permissible in HGPIN. AIDCP is also easily distinguished from invasive cribriform carcinoma by the presence of basal cells in the case of the former. Immunohistochemistry can help to confirm the presence of basal cells in difficult cases.

The most problematic differential diagnosis is its distinction from IDC. Immunohistochemistry for basal cell markers is not helpful in this setting as both entities show a present, albeit in some cases fragmented, basal cell layer. Similarly, PTEN loss and ERG expression are not helpful as AIDCP and IDC have shown a similar pattern of PTEN and ERG expression [10]. The distinction is, thus, based on morphology and the discriminative features are more a matter of quantity, rather than their presence or absence. When architecture is not dense and nuclear atypia is not high enough to make an IDC diagnosis, then the cribriform lesion is called AIDCP. In our experience, AIDCP commonly coexists with IDC in prostatectomy specimens, meaning that adjacent to ducts with typical features of IDC are ducts with an intraductal proliferation that is very similar to, but falls short of, IDC diagnosis. Whether the criteria for IDC need to be loosened in those cases to include all cribriform intraductal proliferations remains to be determined. This, however, has mainly academic interest, as areas with definite IDC are usually present as are areas with invasive carcinoma and the nature (and nomenclature) of the not-typical-for-IDC cribriform formations will not have any significant implications for the patient’s further management.

From a practical standpoint, the diagnosis of AIDCP has important clinical implications in biopsy specimens as, when isolated, this has correlated with an increased probability of the presence of an invasive carcinoma in repeat biopsy [10,13]. For this reason, the presence of isolated AIDP in biopsies should be treated with close surveillance and re-biopsy and should not be considered as HGPIN [7,11,14]—an entity that does not usually justify a biopsy repeat.

## 5. Malignant Cribriform Formations

### 5.1. Intraductal Carcinoma (IDC)

**Figure 3:**
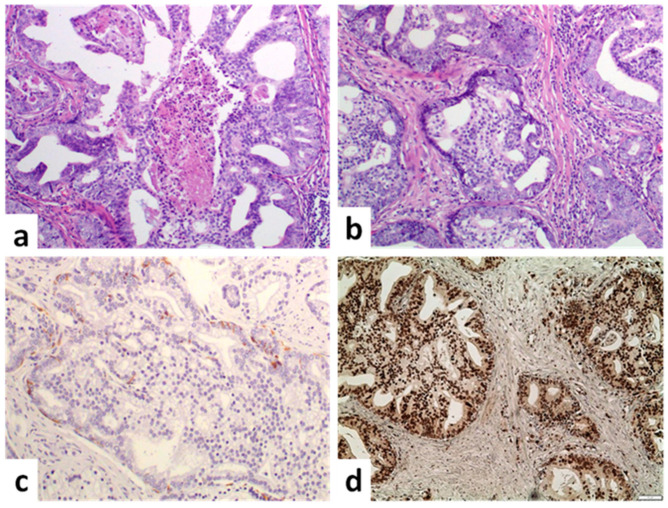
Intraductal carcinoma. (**a**) An expanded gland with cribriform architecture, central necrosis and preservation of basal cells (×100). (**b**) Confluent foci of expanded glands with dense cribriform architecture (×100). (**c**) Basal cell layer is fragmented (basal cell marker high molecular cytokeratin 34βE12) (×100) (**d**) ERG expression is observed in the neoplastic cells (×100) (scale bar is 50 μm).

IDC is characterized by a proliferation of malignant epithelial cells within preexisting prostatic ducts and acini with architectural and/or cytological atypia that exceeds that of high-grade prostatic intraepithelial neoplasia [15]. Thus, by definition, IDC retains the basal cell layer, although it is well known that some IDC foci can have dispersed basal cells due to gland distention. Acini and ducts are usually greatly expanded, and the cells often exhibit remarkable nuclear abnormalities such as enlargement and significant hyperchromasia of the nuclei. Major and minor histological criteria have been used for IDC diagnosis. The major criteria include solid or dense cribriform architecture, significantly enlarged nuclei and non-focal comedonecrosis [16]. The dense cribriform architecture is defined as cells involving >50% of the duct lumen, and the enlarged nuclei were originally defined as ≥6 times the size of the normal cells (Figure 3). However, the later has recently been disputed [17,18] and replaced by marked pleomorphism and nucleomegaly [19]. Minor criteria include irregularly shaped glands with right-angle branching, frequent and easily identifiable mitoses and the existence of two cell populations, one mitotically active at the periphery that exhibits pleomorphism and faint PSA expression, and the other involving quiescent cells in the center with strong PSA expression [20].

IDC is usually accompanied by adjacent invasive carcinoma both in biopsies [21,22] and prostatectomy specimens [22], with the invasive carcinoma usually, but not always, being high-grade and high-stage [23,24]. IDC is associated with adverse pathologic parameters [25] (i.e., tumors with advanced Gleason Score [26], extra prostatic extension [27], seminal vesicle invasion [21], large volume [26] and presence of lymph node metastasis [28]). In addition, the presence of IDCp has been independently correlated with a reduction in progression-free survival in hormone-naïve [29,30], treated [31] and CRPC patients [26,32]. Thus, its presence should be noted in the pathology report in both needle biopsies and prostatectomy specimens.

Even when the accompanying invasive carcinoma is low-grade, the finding of IDC in biopsies is associated with a high probability of upgrading in the subsequent prostatectomy and disease progression if an active surveillance protocol is used [23]. Thus, IDC in biopsies is considered an adverse finding and active surveillance is not recommended, even when the invasive carcinoma is low-grade [19,33,34]. Rarely, IDC may be the sole finding either in biopsies [15,35] or prostatectomy specimens [24,32,36]. Of note, in the majority, albeit not all, of cases with isolated IDC (i.e., not accompanied by invasive carcinoma) in biopsies, a high-grade, high-stage invasive carcinoma will be found in the subsequent prostatectomy [16,35]. In addition, in a minority of them, metastatic disease will be observed. It has been stated that the presence of an IDC unaccompanied by invasive carcinoma in a prostate biopsy is usually the result of non-sampling a coexisting high-grade invasive component [37]. Thus, the presence of IDC in a needle biopsy should, at the least, lead to an immediate biopsy to search for unsampled high-grade prostatic carcinoma, or even to definitive therapy [16]. The clinical implications of IDC (and other cribriform-patterned atypical/malignant lesions—see below) are summarized in Table 2.

Regarding the pathogenesis of IDCp, there are two theories. The first and most prevalent one suggests that IDC results from the spreading of the accompanying invasive high-grade carcinoma into existing non-neoplastic ducts and acini [38]. The adverse pathology and prognosis that IDC is associated with supports the notion that, in the majority of cases, IDC is a late stage of the prostate tumor progression pathway. Indeed, IDC has a genetic profile and genomic instability that is very similar to prostate cancer Gleason 4 and 5 [39] and not lower Gleason Score carcinomas [25]. Loss of *PTEN*, *CDH1*, and *BRCA1* and gain of *MYC*, features of aggressive invasive carcinomas, are genetic abnormalities shared by IDC too [25,39]. Of note, the presence of IDC is used as an indicator to test patients with PCa for germline mutations of genes of proteins involved in DNA repair through homologous recombination (i.e., *BRACA2, BRACA1*) [40,41,42]. The most common chromosomal alteration observed in IDC is the fusion of the *TMPRSS2–ERG* genes, a genetic event that is observed early in the evolution of PCa; when present, it is shared by the adjacent invasive carcinoma [43]. In contrast, when the invasive component is ERG-negative, the associated IDC is also negative. This supports a common clonal origin and indicates that IDC represents an intraductal spread of invasive carcinoma [7,43,44]. The loss of cytoplasmic PTEN expression, a late-onset event occurring in a subclonal population of neoplastic cells during PCa evolution, is another genetic event frequently shared by IDC and the accompanying invasive carcinoma [11,22,25,45,46]. Indeed, previous studies have shown that PTEN-negative HGPIN and IDCp adjacent to invasive cancer are more likely to represent the intraductal spread of malignant cells rather than de novo precancerous lesions [7,43]. The reason why cancer cells make their way inside pre-existing ducts has yet to be explained. Based on the increased prevalence of IDC following therapy and its association with a worse prognosis [31], poor clinical response to therapy [47] and the absence of pathology features of response to therapy in IDC foci [48,49], we may hypothesize that tumor cells find a safe-haven within the pre-existing ducts and away from the tumor stroma; this helps them to better adjust to an ominous microenvironment. Indeed, experiments with patient-derived xenograft models have shown that cells within IDC are able to withstand castration and regenerate the tumor following testosterone restoration [50]. Additional evidence is needed to support this theory and determine whether this also holds true for IDC in untreated tumors.

The second theory regarding IDC pathogenesis states that, at least in some cases, IDC may represent a precursor lesion. The presence of a few cases of isolated IDC even after full embedding of the prostate supports this theory. IDC may reflect an earlier step in the evolution of the metastatic clone [51] as it has been shown that it is more closely related to the lymph node metastases than the adjacent invasive carcinoma [52], and that IDC and adjacent low-grade carcinoma are not genetically related [24]. The latter is based on the observation that, in 15 cases of IDCp with concurrent low-grade invasive carcinoma analyzed with next-generation sequencing and immunohistochemistry for PTEN and ERG, IDC showed activation in the MAPK/PI3K pathway and a discordant PTEN/ERG status with the adjacent low-grade carcinoma [24]. Thus, in this scenario, IDC may represent a precursor lesion that quickly progresses to a high-grade invasive carcinoma and, as a result, is rarely observed alone.

IDC must be differentiated from high-grade PIN, atypical cribriform lesions and invasive carcinoma. Distinction from HGPIN and AIDCP has been discussed in the previous sections. It is well-established that IDC can be a morphologic mimicker of invasive cribriform carcinoma. The identification of basal cells is the most important feature that distinguishes IDC from invasive PCa. The presence of corpora amylacea and branched architecture supports the diagnosis of IDC and is a helpful clue in cases where basal cells are not readily identified [53]. However, in some cases, a distinction between IDC and invasive cribriform pattern carcinoma cannot be identified with certainty based on H&E alone; therefore, immunohistochemistry with basal cell markers may be used [54]. Of note, the basal cell layer in IDC is often fragmented and may not be present in the plane/level of section. Thus, foci without a basal cell layer are morphologically indistinguishable from adjacent foci that have a basal cell layer, which questions our ability to accurately distinguish IDC from an invasive carcinoma even with the use of immunohistochemistry, especially when performed on a single level [55].

A differential diagnosis of IDC also includes an intraductal spread of the urothelial carcinoma. Neoplastic proliferation is within pre-existing ducts in both cases. The presence of longitudinal nuclear grooves is indicative of the urothelial origin of the cells and may be used as a clue to consider urothelial carcinoma, but is present only in low-grade tumors [56]. A co-existing urothelial carcinoma in situ or papillary urothelial carcinoma in the urethra is also indicative that the neoplastic proliferation within the ducts is of urothelial origin, but these findings may only be appreciated in prostatectomy specimens and, even then, they may not always be present due to epithelial denudation. Immunohistochemistry can help in difficult cases, as IDC expresses PSAP and PSA; whereas urothelial-specific markers (p63, uroplakin, GATA3) are expressed in urothelial carcinomas [8].

Both the International Society of Urological Pathology (ISUP) and the Genitourinary Pathology Society (GUPS), the two international organizations relative to uropathology, agree that IDC is an adverse prognostic factor, that its presence should be noted on the pathology report and that its clinical implications should be commented upon [19,33]. In addition, they both recommend against grading isolated (pure) IDC. However, there are conflicting recommendations regarding the grading of IDC when accompanied by invasive carcinoma and of how to best communicate the adverse clinical implications of IDC to clinicians. ISUP recommends incorporating IDC in Gleason Score/PGG by assigning it a Gleason Grade of 4 (or 5 when comedo necrosis is present) [33], whereas GUPS does not support IDC incorporation in Gleason Score/PGG and only recommends reporting the IDC component as a comment in the pathology report [19]. Table 3 summarizes the current guidelines regarding the grading of IDC and other cribriform-patterned lesions.

There are arguments in favor of both approaches. The distinction of IDC from invasive cribriform carcinoma can be difficult on H&E alone and would require immunohistochemical staining in some cases, creating an extra burden for the pathology laboratory. In addition, as discussed above, the discrimination of IDC from an invasive carcinoma with a cribriform pattern may not be possible even when immunohistochemistry is used. Furthermore, IDC is included in the estimation of tumor extent; thus, it seems logical to include it in PCa grading. Finally, as mentioned above, the presence of IDC is an adverse parameter in patients, even when associated with PGG1 PCa [23]; in addition, excluding IDC from the Gleason Score can underestimate the patient’s risk. In agreement with this, studies have shown that a grading system that incorporates IDC better describes patient prognosis [61,62] and that not only the presence, but also the amount of IDC is associated with adverse prognostic parameters, further supporting its incorporation in grading in a quantitative fashion [54].

On the other hand, IDC is sometimes, albeit rarely, a precursor lesion and has been shown to be a genetically different form of adjacent low-grade carcinoma, indicating that, in these cases, the incorporation of IDC to PGG would, in fact, overestimate the patient’s risk. The distinction of IDC from an invasive carcinoma is not required in all cases; it is only required in cases where a change in PGG will be observed depending on the % of IDC. Studies with routine cases have shown that the inclusion or exclusion of IDC in tumor grading results in a global Grade Group shift in <2% of cases [53,54,63]. However, in a minority of cases, the change in PGG was significant (>2 points) when IDC is incorporated in the Gleason Score [54,63], usually due to the presence of comedonecrosis.

The issue of whether IDC should be included in grading is far from resolved. Even though there are studies that support its incorporation in grading, prospective studies with survival as the endpoint are lacking. In addition, the effect of a grading system where IDC is taken into account may be missed because of the far larger number of cases where the grade is not altered regardless of the presence and amount of IDC. Studies that focus on cases in which the grade would change when the IDC is incorporated and with survival as the endpoint would be informative in terms of which grading system has the most clinical relevance.

### 5.2. Invasive Acinar Carcinoma with Cribriform Pattern

The acinar adenocarcinoma is the main histologic type of prostate carcinoma, accounting for the majority of all primary carcinomas of the prostate [15]. Prostate cancer grading was originally proposed in 1974 by Donald Gleason [64] and, with important refinements across the years [57,58] and the recent adoption of the Prognostic Grade Grouping nomenclature [65], it remains one of the most important prognostic parameters in PCa. Among the Gleason Grades (GG), GG4 is the most heterogeneous pattern both morphologically and clinically. Architecturally, four growth patterns are recognized within GG4: ill-formed glands, fused glands, and glomeruloid and cribriform patterns. Clinically, numerous studies reported a correlation between the cribriform pattern with adverse pathologic features and adverse outcomes compared to the other GG4 patterns [66]. In particular, tumors with a cribriform pattern have adverse pathologic parameters more frequently than tumors without this pattern [39,54,67,68] and the patients show earlier PSA recurrence [69,70,71,72], more frequent metastasis [72,73] and shorter disease-specific survival [73]. Its clinical significance is best appreciated in GS 7 tumors. Patients with a biopsy of GS 7 PCa without the cribriform pattern have a similar prognosis to those with a GS 6 tumor [39]. In line with the clinical and pathologic observations, unfavorable molecular characteristics are observed in tumors with a cribriform pattern (and intraductal carcinoma) which is similar to the alterations observed in aggressive prostate cancer, including increased genomic instability [74,75], oncogene amplifications, and tumor suppressor genes copy number alterations and point mutations [74]. Thus, the presence of a cribriform pattern in a GS7 tumor on the biopsy is considered a contraindication for active surveillance. Both GUPS and ISUP agree that the cribriform invasive carcinoma should be assigned a Grade 4 (or 5 when comedo necrosis is present) [57]. In addition to being factored in grade, a comment regarding the presence and clinical significance of the cribriform pattern should be included in the report in both biopsies and prostatectomy specimens [19].

**Figure 4:**
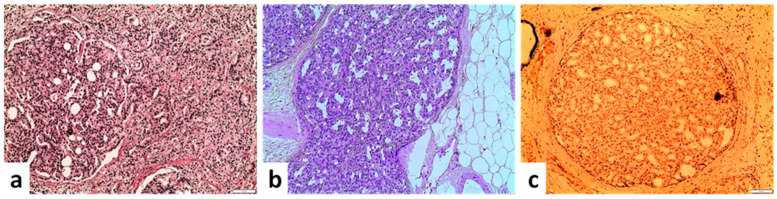
Invasive cribriform acinar carcinoma. (**a**) Merging of cribriform structure to clearly invasive carcinoma (×100). (**b**) Complex cribriform structure invading in the periprostatic adipose tissue verifies the invasive nature of the structure (×100). (**c**) Absence of staining for basal cell marker verifies the invasive nature of these cribriform glandular structure (Gleason pattern 4) (Immunohistochemistry with the basal cell marker high molecular cytokeratin 34βE12) (×100) (scale bar is 50 μm).

Cribriform acinar carcinoma includes both small, rounded cribriform glands characterized by a continuous proliferation of cells with intermingled lumina [57,76,77] and larger irregular cribriform glands (>3 times the size of normal glands). Cytologic features of atypia typical of PCa such as nuclear enlargement, anaplasia and prominent nucleoli are also observed (Figure 4). A basal cell layer is typically lacking. It is also worth mentioning that the cribriform pattern shows a higher inter-observer reproducibility among pathologists in comparison with the other Gleason pattern 4 variants [78].

Mimickers of invasive cribriform carcinoma include IDC and ductal carcinoma. The distinction of cribriform pattern carcinoma from IDC is analyzed above. The distinction from ductal carcinoma is reported in the next section. Finally, less frequently, invasive cribriform carcinomas need to be distinguished from urothelial carcinoma involving the prostate, as gland-like lumina may be observed in the nests of urothelial carcinoma. In some instances, the cytologic characteristics of the cells (round cells with nucleoli and limited pleomorphism versus elongated nuclei with grooves) or the presence of an in situ/papillary component versus other patterns of PCa may be indicative of urothelial or prostate carcinoma, respectively. In difficult cases, the immunohistochemistry for prostate and urothelial-specific markers may be performed as mentioned in the IDC section.

The pathogenesis of cribriform pattern carcinoma remains obscure. A molecular profile unique to cribriform patterned invasive carcinoma has been shown by numerous studies (reviewed in [79]). These molecular alterations frequently involve pathways associated with aggressive tumor characteristics (i.e., MYC, MAPK, DNA repair, integrin signaling) and may account for the aggressive behavior of the cribriform pattern. However, these alterations may neither be the result nor the consequence of cribriform pattern, as they are not cribriform-specific. One of the most characteristic histologic features of the cribriform pattern in invasive carcinomas is that the majority of the intraglandular cells lack contact with the stroma [80,81]. In contrast, in Gleason Grade 3 and other patterns of Gleason Grade 4 (fused glands, poorly formed glands), most of the tumor cells are in contact with the stroma [81]. This feature leads us to hypothesize that, similar to IDC, tumor cells find a safe haven within cribriform structures, away from the potentially harmful effects of the stroma. In addition, hypoxia that is higher within cribriform formations [75], probably as a result of the limited contact of the cells to the vessel-containing stroma [81], and the effects of a specific type of tumor-associated fibroblast found around cribriform structures that is distinct from fibroblasts associated with other morphologic patterns [82], may account for the aggressive behavior of cribriform-patterned carcinoma.

Numerous studies have shown that tumors with a cribriform (and intraductal carcinoma) pattern have distinct genetic and epigenetic signatures [41,71,79,83] compared to tumors without these aggressive patterns. However, head-to-head comparisons of gene expression between cribriform and non-cribriform areas within the same tumor are very sparse and mostly performed using immunohistochemistry. Proteins reported to be upregulated in cribriform areas (compared to other GG4 patterns) include EGFR [84] and the cell proliferation marker ki67 [85]. In addition, CD44 downregulation [84] and PTEN [86] loss are more common in cribriform compared to non-cribriform areas. Integrins have also shown distinct patterns of expression in cribriform and non-cribriform areas [87], which is not surprising given that these molecules are the cells’ sensor of the extracellular environment [88]. It has been hypothesized that a prostate cancer ‘nimbosus’ (gathering of stormy clouds in Latin) [75], characterized by the gathering of multiple unfavorable events (i.e., hypoxia [75] genetic instability [74,75] and various adverse molecular alterations [75,79]), happens in cribriform carcinoma and probably accounts for its aggressiveness.

### 5.3. Ductal Carcinoma

Ductal carcinoma is an uncommon and aggressive morphologic type of prostate cancer. It is found in 1–6% of PCa cases, usually mixed with high-grade acinar adenocarcinoma [89] and represents only 1% of prostate carcinomas in its pure form [15,45]. It typically presents as a mass within the prostatic urethra and large periurethral prostatic ducts, although tumors morphologically similar to ductal carcinoma may be observed in the peripheral zone too [90]. When located in the periurethral area, it presents with hematuria and obstructive symptoms.

**Figure 5:**
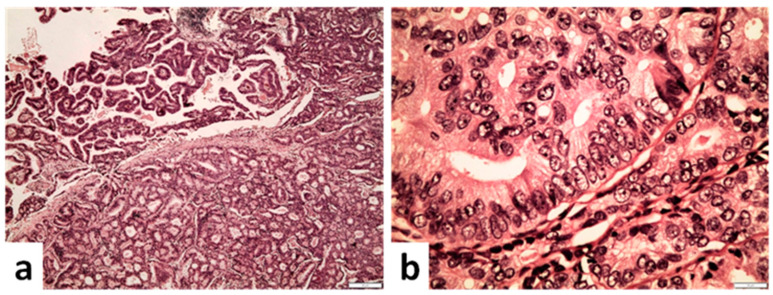
Ductal carcinoma of the prostate. (**a**) Papillary and cribriform architecture coexisting in a ductal adenocarcinoma of the prostate gland (×40). (**b**) Columnar tumor cells within a cribriform structure compatible with ductal carcinoma (×400) (scale bar is 50 μm).

Clinically, ductal carcinoma is associated with worse pathologic parameters [89] and is an independent indicator of worse biochemical-free [90,91], metastasis-free [92] and overall survival [93] compared to acinar carcinoma. Morphologically, ductal carcinoma may have various patterns, usually more than one within the same case, the most characteristic being a papillary pattern with true fibrovascular cores lined by tall pseudostratified columnar cells (Figure 5) [94]. A glandular, cribriform and solid pattern may also be observed, but in these cases, a distinction from acinar carcinoma is more difficult and relies on the identification of the characteristic cellular features [94]. The neoplastic cells are tall, columnar and pseudostratified, resembling endometrial adenocarcinoma, hence why it was originally thought to be of mullerian origin [95]. Significant nuclear atypia and high mitotic activity are also frequently observed. The cytoplasm is typically amphophilic, although it can be clear. Immunohistochemical staining for basal cell markers may be focally positive [96], as ductal carcinoma is well known for its ability to involve and expand pre-existing glands.

Despite the morphologic and phenotypic differences between ductal and acinar carcinoma, few molecular differences have been depicted between the two entities [97] and are better appreciated when pure forms of ductal adenocarcinoma are examined. Genetic alterations in genes associated with chromatin modification, the PI3K and APC pathway, cell cycle/apoptosis, and transcription regulation, similar to advanced and metastatic acinar prostate cancer, have been shown in ductal carcinoma [98]. In addition, an intrinsic upregulation of androgen-resistance pathways [93] and frequent DNA repair gene aberrations [99,100] have been described in ductal adenocarcinoma and may account for its aggressive clinical behavior.

To account for its aggressive behavior, ductal carcinoma is assigned a Gleason Grade 4 [58]. Similar to acinar carcinoma, a Gleason Grade 5 is given when comedo necrosis is present [45]. Due to its more favorable clinical behavior [59], PIN-like ductal adenocarcinoma should be assigned a Gleason Grade 3.

The cribriform pattern of ductal carcinoma needs to be distinguished from intraductal, and invasive acinar cribriform carcinoma. The typical cellular features, i.e., tall columnar pseudostratified cells may be difficult to appreciate in this pattern, making the distinction more difficult, especially in a biopsy specimen [94]. The presence of true papillae, slit-like lumina and elongated cells are in favor of ductal carcinoma, whereas punched-out lumens and rounded nuclei are typically observed in invasive acinar and intraductal carcinoma [94,101]. Immunohistochemistry is not particularly helpful. PTEN loss and ERG expression have been described less frequently in ductal carcinoma compared to acinar carcinoma [102]; however, both markers may be observed in ductal carcinoma; hence, they cannot be used as a reliable factor for differential diagnosis between the two entities. Similarly, a lack of basal cell markers is in favor of ductal carcinoma versus intraductal carcinoma; however, the opposite is not true, as ductal carcinomas may spread within prostatic ducts [96].

### 5.4. Basal Cell Carcinoma

Basal cell carcinoma is an unusual type of prostate cancer composed of basal cells [15]. Morphologically, it shows an adenoid cystic carcinoma-like pattern, with irregular cribriform formations containing mucin or basement membrane-like material within the lumina. The stroma is usually desmoplastic. Cytologically, the nuclei are large and hyperchromatic with scant cytoplasm. Nucleoli may or may not be prominent. Basal cell carcinoma has been considered an indolent tumor; however, almost half of the cases are associated with high-risk features and local recurrence [103]. Immunohistochemically, the tumor is positive for basal cell makers p63 and 34bE12 and negative for luminal markers PSA and PAP [104]. CK7 can be positive and AMACR staining is weak to negative [15]. At the molecular level, basal cell carcinoma shows a basal cell gene signature [105] with genetic aberrations that are distinct from acinar adenocarcinoma, i.e., frequent EGFR overexpression and absence of ERG rearrangements [103]. Differential diagnoses from other cribriform lesions of the prostate are based on the characteristic cytologic and architectural features mentioned above, as well as the distinct immunohistochemical profile. The most important distinction is from basal cell hyperplasia, a benign condition with excellent prognosis. The presence of an adenoid cystic carcinoma-like pattern and anastomosing, variably sized and irregularly shaped nests and tubules are in favor of basal cell carcinoma [15]. A widely invasive pattern in between normal prostatic acini and the presence of extraprostatic extension are virtually diagnostic of basal cell carcinoma (versus basal cell hyperplasia). Bcl-2 expression and high ki67 are more commonly observed in basal cell carcinoma compared to basal cell hyperplasia [106] and can be of use in difficult cases.

## 6. Conclusions

**Figure 6:**
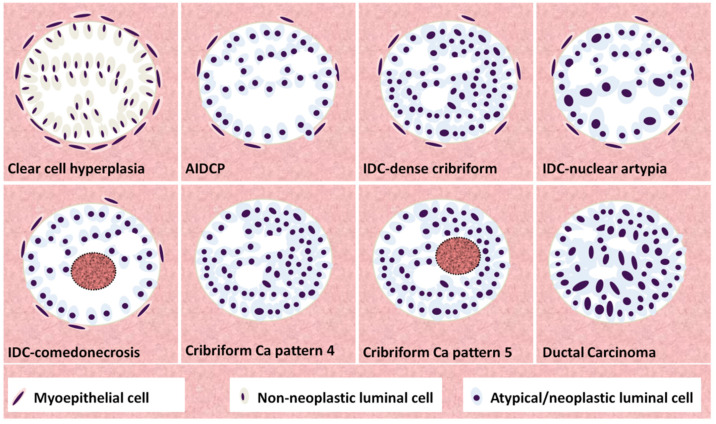
Graphical illustration of the various entities with cribriform morphology. Clear cell hyperplasia is characterized by a loose cribriform proliferation of clear cells without atypia. In atypical intraductal cribrifrom proliferation (AIDCP), atypical cells proliferate within a duct lumen (note the myoepithelial cells at the periphery); however, the architecture is loose, and the cells take up <50% of the surface of the lumen. In intraductal carcinomas (IDC), three patterns may be observed (alone or in combination): dense cribriform (>50% of the surface) or even solid (not shown) proliferation of atypical cells within the duct lumen (note the presence of myoepithelial cells, albeit they may be sparse in some cases) and loose architecture, but with either nuclear atypia or comedonecrosis. In cribriform carcinoma myoepithelial cells are missing (cribriform carcinoma is assigned a Gleason Grade 4). However, a Gleason Grade 5 is assigned if comedonecrosis is present (note again the lack of myoepithelial cells, consistent with invasive carcinoma). In ductal carcinoma, lumina are slit-like (instead of round) and cells are columnar. Myoepithelial cells are usually absent (although intraductal spread may be observed, not shown here).

Overall, understanding the distinct nature of each cribriform lesion is fundamental for rendering the correct diagnosis and ensuring accuracy in clinical decision-making, prognosis prediction and personalized risk stratification of patients. In this review, we summarized the recent literature on the differential diagnosis of all neoplastic and non-neoplastic cribriform formations and explained how cribriform architecture could alter management decisions for prostate cancer patients. All the data and information collected in the review aim to help the pathologist deal with challenging and controversial cases of cribriform prostate patterns in the daily laboratory work practice. A graphical illustration of the various cribriform patterned lesions is shown in Figure 6. The goal is to make the correct diagnosis so as the patient can receive the appropriate clinical management and follow-up.

## Figures and Tables

**Table 1:** Summary of differential diagnosis of entities with cribriform morphology within the prostate gland.

Entity	Architecture	Cytologic Features	Basal Cell Layer	ERG Expression	PTEN Loss
Benign cribriform glands	Complex epithelium with cribriform pattern and epithelial bridges located in the central zone	High columnar stratified epithelium, granular cytoplasm, small round nuclei without cytologic atypia or prominent nucleoli	Intact	−	−
Basal cell hyperplasia	Nodular lesion, within the transitional zone	Scant cytoplasm, hyperchromatic nuclei without cytologic atypia	The lesion involves basal cells	−	−
Clear cell cribriform hyperplasia	Variant of BPH, medium and large sized acini with cribriform morphology	Pale to clear cytoplasm, nuclei lack cytologic atypia or prominent nucleoli	Intact	−	−
HGPIN	Normal-sized acini with tufting, micropapillary, or flat growth pattern and without expansion of glands	Cytologic atypia, nuclear enlargement and hyperchromasia with prominent nucleoli, no necrosis	Preserved (can be fragmented)	−/+	−
AIDCP	Loose cribriform lumen-spanning architecture	Moderate nuclear atypia, absence of necrosis, insufficient to meet the criteria for IDC	Preserved (can be fragmented)	+/−	Identified
Intraductal carcinoma	Greatly expanded glands with cribriform/solid growth	Nuclear atypia (nuclear enlargement, hyperchromatic nuclei) may be present	Preserved (can be fragmented)	+/−	Identified
Invasive cribriform acinar carcinoma	Continuous proliferation of cells with intermingled lumina	Nuclear atypia (prominent nucleoli, hyperchromasia)	Absent	+/−	Identified
Ductal carcinoma	Papillary, solid and cribriform growth pattern	Tall columnar cells, nuclear atypia, mitotic figures	Usually absent, may be present	−/+	Identified
Basal cell carcinoma	Irregular cribriform formations containing mucin or basement membrane-like material within the lumina, desmoplastic reaction	Hyperchromatic large nuclei with scant cytoplasm	The entire process involves basal cells	−	Identified

**Table 2:** Clinical implications of malignant cribriform-patterned lesions in pathology reports.

Cribriform Lesion	Clinical Implication
Atypical intraductal cribriform proliferation (isolated in biopsy)	Increased probability of invasive carcinoma in subsequent biopsy Recommendation for close surveillance and re-biopsy
IDC	Higher prevalence of homologous DNA repair recombination repair defects Germline testing may be considered
IDC with invasive carcinoma PGG1/2 in biopsy	High probability of upgrading and/or upstaging in subsequent prostatectomy Recommendation for definite treatment (active surveillance not recommended)
IDC without invasive carcinoma in biopsy	High probability of invasive carcinoma of high-grade/-stage in subsequent prostatectomy Recommendation for immediate re-biopsy or definite treatment
Cribriform pattern	Higher prevalence of DNA repair mechanisms defects Germline testing may be considered
Cribriform pattern in carcinoma PGG 2 in biopsy	Poor prognostic factor Recommendation for definite treatment (active surveillance not recommended)
Ductal carcinoma	Higher prevalence of DNA repair mechanisms defects Germline testing may be considered

**Table 3:** GUPS and ISUP guidelines regarding the grading of cribriform-patterned lesions.

	GUPS [19]	ISUP [33]
Isolated IDC	No grade assigned	No grade assigned
IDC with invasive carcinoma	IDC not included in grading	IDC graded as pattern 4 (or 5 when comedonecrosis is present)
Comment on IDC clinical significance in pathology report	Recommended	Recommended
Cribrifrom	Grade as patern 4 (based on the ISUP 2014 reccomendations) [57]	
Report the presence of cribriform pattern carcinoma	Yes (biopsies and radical prostatectomies) Comment on the clinical significance	Yes (biopsies and radical prostatectomies)
Ductal carcinoma	Grade as pattern 4 (based on the ISUP 2005 reccomendations) [58] Grade as pattern 3 if PIN-like ductal [59] and pattern 5 if comedonecrosis present [60]	
